# Bleomycin-Induced Lung Injury Increases Resistance to Influenza Virus Infection in a Type I Interferon-Dependent Manner

**DOI:** 10.3389/fimmu.2021.697162

**Published:** 2021-08-18

**Authors:** Sang-Uk Seo, Jae-Hyeon Jeong, Bum-Seo Baek, Je-Min Choi, Youn Soo Choi, Hyun-Jeong Ko, Mi-Na Kweon

**Affiliations:** ^1^Department of Microbiology, College of Medicine, The Catholic University of Korea, Seoul, South Korea; ^2^Laboratory of Microbiology and Immunology, College of Pharmacy, Kangwon National University, Chuncheon, South Korea; ^3^Department of Biomedical Sciences, Seoul National University College of Medicine, Seoul, South Korea; ^4^Wide River Institute of Immunology, Seoul National University College of Medicine, Hongcheon, South Korea; ^5^Department of Life Science, College of Natural Sciences, Hanyang University, Seoul, South Korea; ^6^Mucosal Immunology Laboratory, Department of Convergence Medicine, University of Ulsan College of Medicine/Asan Medical Center, Seoul, South Korea

**Keywords:** acute lung injury, pulmonary fibrosis, influenza virus, plasmacytoid dendritic cells, type I interferon

## Abstract

Acute lung injury (ALI) results in acute respiratory disease that causes fatal respiratory diseases; however, little is known about the incidence of influenza infection in ALI. Using a ALI-mouse model, we investigated the pro-inflammatory cytokine response to ALI and influenza infection. Mice treated with bleomycin (BLM), which induces ALI, were more resistant to influenza virus infection and exhibited higher levels of type I interferon (IFN-I) transcription during the early infection period than that in PBS-treated control mice. BLM-treated mice also exhibited a lower viral burden, reduced pro-inflammatory cytokine production, and neutrophil levels. In contrast, BLM-treated IFN-I receptor 1 (IFNAR1)-knockout mice failed to show this attenuated phenotype, indicating that IFN-I is key to the antiviral response in ALI-induced mice. The STING/TBK1/IRF3 pathway was found to be involved in IFN-I production and the establishment of an antiviral environment in the lung. The depletion of plasmacytoid dendritic cells (pDCs) reduced the effect of BLM treatment against influenza virus infection, suggesting that pDCs are the major source of IFN-I and are crucial for defense against viral infection in BLM-induced lung injury. Overall, this study showed that BLM-mediated ALI in mice induced the release of double-stranded DNA, which in turn potentiated IFN-I-dependent pulmonary viral resistance by activating the STING/TBK1/IRF3 pathway in association with pDCs.

## Introduction

Acute respiratory distress syndrome (ARDS) is a fatal pulmonary disease characterized by pulmonary fibrosis, hypoxemia, and infectious complications that are triggered by acute lung injury (ALI) (1–4). Approximately 150,000 individuals in the United States are diagnosed with ARDS annually with a survival rate of approximately 40% over the 20 years after diagnosis (1, 5). ALI can also be caused by exposure to silica and by pulmonary viral infections, and the anticancer drug bleomycin (BLM) has been used to develop a murine model of ALI (6–8).

Injury to lung epithelial cells results in the release of DNA fragments that activate the cyclic GMP-AMP synthase (cGAS)-stimulator of IFN genes (STING) pathway to stimulate type I interferon (IFN-I)-dependent immune responses (6, 9, 10). IFN-I is a crucial cytokine that regulates the response of the immune system to viral infection (11). IFN-I binds to its receptor (IFNAR) and induces the expression of IFN-stimulated genes (ISGs) such as Mx1 and ISG15, which inhibit viral replication in host cells and activate immune cells for effective viral clearance (12).

IFN-I is produced by various cell types in lung tissues, including alveolar epithelial cells, alveolar macrophages, and dendritic cells (DCs) (13). Plasmacytoid DCs (pDCs) recognize self-DNA and viruses and robustly produce IFN-I that controls viral infection *via* TLR7-, TLR9-, and cGAS-dependent pathways (14, 15). In addition, pDCs sense apoptosis- or necrosis-derived nucleotides and produce IL-10 and IFN-I to maintain tolerance (16). By sensing released host DNA, pDCs repair damaged epidermis in an IFN-I-dependent manner (17).

Patients with ARDS are reported to be vulnerable to bacterial complications, but the incidence of respiratory virus infection in patients with ARDS remains unknown (18). Using a mouse model, we found that BLM-induced ALI releases self-DNA that activates the STING/TBK1/IRF3 pathway to develop pulmonary virus resistance in an IFN-I-dependent manner. Collectively, BLM-induced ALI developed an antiviral environment in the lungs in an IFNAR1- and pDC-dependent manner *via* prompting by self-DNA.

## Materials and Methods

### Animal Model

Specific pathogen-free 8-week-old female C57BL/6 mice, weighing 18–20 g, were purchased from Charles River Laboratories (Orient Bio Inc., Sungnam, Korea). IFNAR1^-/-^ mice were purchased from B&K Universal Ltd. (Hull, U.K.). All mice were bred and housed in the Animal Laboratory Center of Kangwon University. To induce lung injury and pulmonary fibrosis, mice were anesthetized by injecting a mixture of 100 μL of ketamine (25 mg/mL) and xylazine (2 mg/mL), and then intranasally injected with 1 mg/kg of BLM (Merck) dissolved in 30 μL PBS or PBS alone. On day 14, the mice were anesthetized and infected i. n., with a sub-lethal (1 × 10^3^ plaque forming units [PFU]) or lethal (1 × 10^5^ PFU) dose of A/PR/834 (A/PR8) in 20 μL PBS. DNase I (100 μg/mouse, Roche) and αIFNAR1 (100 μg/mouse, BioXcell) were administered after BLM treatment.

### Influenza Virus

A/Puerto Rico/8/34 influenza virus (A/PR8) incubated in allantoic fluid was kindly provided by Prof. Baik Lin Seong at Yonsei University.

### Isolation of Bronchoalveolar Lavage Fluid

The mice were euthanized, their tracheas were exposed, and a catheter was inserted to flush the lungs with 1 mL of PBS. Bronchoalveolar lavage fluid (BALF) samples were centrifuged at 500× *g* for 5 min to collect the cells. The supernatants were centrifuged at 14,000 rpm for 1 min to completely remove the cells.

### Isolation of Total Lung Immune Cells

Lungs removed from the sacrificed animals were diced using scissors. Tissue samples were suspended in 5 mL of digestion buffer containing RPMI-1640 (Gibco), 2% heat-inactivated fetal bovine serum (Gibco), 10 mM HEPES (Gibco), 1% penicillin-streptomycin (Gibco), 400 U/mL of collagenase D (Worthington), and 0.01 mg/mL DNase I (Roche), and minced using C-tube (MACS). The samples were incubated for 1 h at 37°C with shaking at 200 rpm. The cells were collected in PBS containing 10 mM EDTA and centrifuged at 500× *g* for 5 min. The pellet was resuspended in 1 mL RBC lysis buffer (Invitrogen) and incubated for 1 min at room temperature (RT, 20°C). The cells were then washed with PBS before use.

### *In Vitro* Assays

Levels of TNF-α, IFN-γ, IL-6, CCL2, IL-12p40 (Thermo Fisher), and CXCL1 (R&D Systems) were measured using enzyme linked immunoassay (ELISA) kits according to the manufacturer’s instructions. Apoptotic cells in BALF were analyzed using the Annexin V Apoptosis Detection Kit (Biogems) according to the manufacturer’s instructions. Double-stranded DNA (dsDNA) in BALF was measured using a DNA quantification assay kit (Abcam) according to the manufacturer’s instructions.

### mRNA Quantification

Total mRNA was extracted from the lung using TRIzol (Invitrogen), and cDNA was synthesized using reverse transcriptase (Promega) according to the manufacturer’s instructions. The cDNA sequences were amplified using SYBR Green pre-MIX (Promega). Target mRNA levels were normalized relative to *GAPDH* mRNA expression. Primers used for quantitative reverse transcription polymerase chain reaction included those for *GAPDH* (forward, 5′-CAGCCTCCAGATCATCAGCA-3′, reverse, 5′-TGTGGTCATGAGTCCTTCCA-3′); *Ifna4* (forward, 5′-TGATGAGCTACTACTGGTCAGC-3′, reverse, 5′-GATCTCTTAGCACAAGGATGGC-3′); and *Ifnb1* (forward, 5′-CAGCTCCAAGAAAGGACGAAC-3′, reverse, 5′-GGCAGTGTAACTCTTCTGCAT-3′). All primers were synthesized by Macrogen Inc. (Seoul, South Korea).

### Plaque Assay

Whole lungs were removed from each mouse, weighed, and homogenized in a 2 mL tube containing PBS and plastic beads. The tissue samples were centrifuged at 12,000 rpm for 5 min, and the supernatants were collected and stored at -80°C until use. A549 cells were cultured in DMEM (Corning) supplemented with 1× antibiotic-antimycotic (A/A) reagent (Gibco) and 10% heat-inactivated fetal bovine serum, were seeded at 1 × 10^6^ cells per well in 6-well plates (Corning) 1 day before analysis. Samples were serially diluted in 1 mL of DMEM supplemented with 1% A/A and incubated with PBS-washed cells at 37°C and 5% CO_2_ for 1 h. The cells were washed with PBS and overlaid with DMEM supplemented with 1% A/A and 1% agarose. After incubation for 5 days at 37°C and 5% CO_2_, the overlay was removed, and the cells were fixed in 4% formalin overnight at RT. Fixed cells were stained with 1% crystal violet for 30 min at RT and washed three times for 20 min each with 1% acetic acid, and plaques were counted.

### Western Blot

Lung tissue was lysed with protein extraction solution (iNtRON) containing 1× protease inhibitor cocktail (Sigma) and 1× phosphatase inhibitor cocktail (GenDEPOT). The lysates were centrifuged at 4,000 rpm for 10 min, and the supernatants were collected. Protein concentrations in the supernatants were measured using a BCA protein assay kit (ThermoFisher). Samples (30 μg) were loaded onto 10% polyacrylamide gels, which were electrophoresed at 120 V for 90 min using a Mini-PROTEAN Tetra Cell (Bio-Rad). The proteins were transferred to nitrocellulose membranes using Trans-Blot SD (Bio-Rad) at 250 mA for 90 min. The membranes were blocked with 5% (w/v) skim milk in 1× TBS-T (20 mM Tris base, 150 mM sodium chloride, and 0.05% Tween-20, pH 7.6) prior to incubation with mouse anti-mouse antibody or in 1× TBS-T containing 5% BSA prior to incubation with rabbit anti-mouse antibody. The membranes were incubated with primary antibodies, including rabbit anti-STING (#13647, Cell Signaling), rabbit anti-phospho-STING (#85735, Cell Signaling), rabbit anti-TBK1/NAK (#3013, Cell Signaling), rabbit anti-phospho-TBK1/NAK (#5483, Cell Signaling), rabbit anti-IRF3 (#4302, Cell Signaling), rabbit anti-phospho-IRF3 (#4947, Cell Signaling), and mouse anti-β-actin (sc-47778, Santa Cruz) according to the manufacturer’s instructions. The membranes were washed three times for 20 min each with 1× TBS-T at RT and incubated for 2 h at RT with goat-anti-rabbit-HRP-conjugated antibody, diluted 1:2500 in 1× TBS-T containing 2.5% BSA, or mouse-anti-mouse-HRP-conjugated antibody, diluted 1:5000 in 1× TBS-T containing 5% skim milk. The membranes were washed three times for 20 min each with 1× TBS-T at RT. Binding was detected using chemiluminescent reagents (G-BIOSCIECNE) and captured with PXi gel doc system (Biorad). Arbitrary units were determined using ImageJ software (NIH).

### Flow Cytometry

The collected lung immune cells were incubated with anti-CD16/CD32 (2.4G2) and in the dark for 30 min at 4°C with various combinations of fluorescent-conjugated antibodies against CD11c (N418), CD11b (M1/70), F4/80 (BM8), Ly6C (HK1.4), Ly6G (1A8), PDCA1 (HM1.2), CD45 (30-F11), CD3 (145-2C11), CD4 (RM4-5), CD8 (53-6.7), CD19 (6D5), and NK1.1 (PK136) (all from BioLegend). DAPI or 7-ADD were used to separate live and dead cells. The cells were sorted using a FACS Verse flow cytometer (BD Bioscience), and the data were analyzed with FlowJo version 10.5.3 software (BD Bioscience) (**Supplementary Figure 1**).

### *In Vivo* Cell Depletion

Mice were injected intraperitoneally three times with 300 μg of αNK1.1 (PK136) and 300 μg of αCD8 (Lyt2.1) 7 days before A/PR8 infection. Mice were subsequently injected intraperitoneally with 80 μg of αCD317 (PDCA-1) 1 day before and 6 and 13 days after BLM treatment and 3 days after A/PR8 infection. All depletion antibodies were purchased from BioXcell.

## Results

### BLM Treatment Increases the Resistance of Mice to Influenza Virus Infection

**Figure 1:**
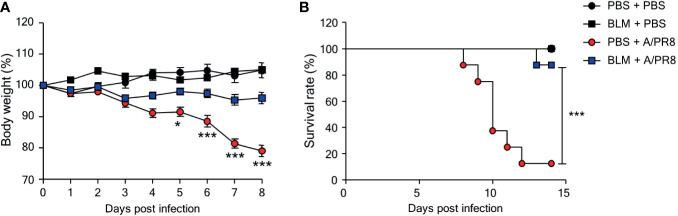
Bleomycin (BLM) treatment increases resistance to influenza virus infection. Mice were intranasally administered BLM or PBS 14 days before infection. **(A)** Mice were intranasally infected with sub-lethal doses and body weights were monitored for 7 days (●, n = 2; ■, n = 3; ●, n = 8; ■, n = 11). **(B)** Mice were intranasally infected with lethal doses, and survival rates were monitored (●, n = 4; ■, n = 4; ●, n = 8; ■, n = 8). Data are expressed as mean ± SEM and representative of 2 independent experiments. Statistical analyses of body weight and survival rates were performed using Two-way ANOVA and the Mantel-Cox test, respectively (^*^ *p* < 0.05, ^***^ *p* < 0.001).

**Figure 2:**
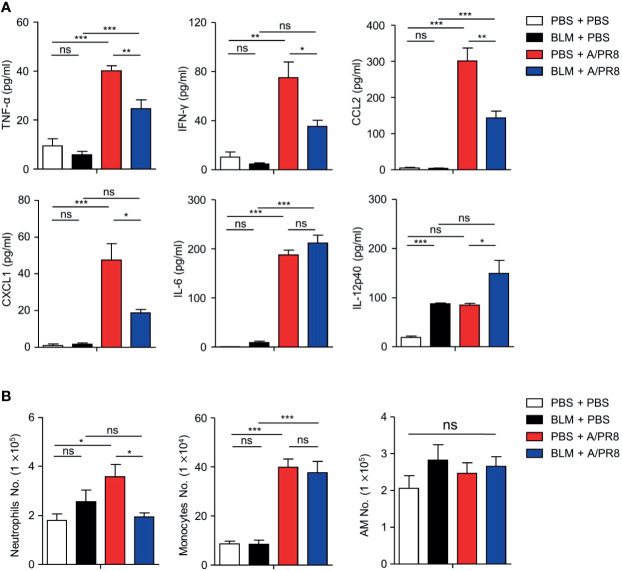
Proinflammatory cytokines and cells were decreased in BLM-treated mice after influenza virus infection. BLM-treated mice were infected intranasally with sub-lethal doses and sacrificed 3 days later. **(A)** BALF was extracted and TNF-α, IFN-γ, CCL2, CXCL1, IL-6, and IL-12p40 concentrations were measured by ELISA (□, n = 4; ■, n = 4; ■, n = 8; ■, n = 7). **(B)** Absolute numbers of neutrophils, monocytes, and alveolar macrophages (AMs) in the lungs were analyzed by FACS (□, n = 9; ■, n = 6; ■, n = 10; ■, n = 9). Data are expressed as ± SEM and representative of 2 independent experiment. Statistical analyses of column were performed using One-way ANOVA (*p < 0.05, **p < 0.01, ***p < 0.001). ns, not significant.

To test whether BLM-induced lung injury can affect susceptibility to viral infection, female B6 mice were treated with BLM (1 mg/kg) or PBS and housed for 14 days until their body weight returned to normal. Groups of mice were then infected with sub-lethal doses (1 × 10^3^ PFU) of A/PR8; body weight was then monitored daily for 8 days. A/PR8 infection reduced the mean body weight by approximately 20% in PBS-treated mice compared with a reduction of less than 5% in BLM-treated mice (**Figure 1A**). To test whether BLM also induced resistance to a lethal dose of A/PR8, mice were infected with 1 × 10^5^ PFU of A/PR8, and the survival of mice was monitored for 14 days. BLM-treated mice showed a higher survival rate (87.5%) than PBS-treated mice (12.5%) (**Figure 1B**), indicating that BLM induces resistance to both sub-lethal and lethal doses of A/PR8. A/PR8 infection markedly increases the production of pro-inflammatory cytokines and cellular infiltrates, and consequently results in severe pulmonary inflammation with cytokine storm (19). To test whether BLM treatment could reduce A/PR8-mediated pulmonary inflammation, mice treated with BLM or PBS were infected with sub-lethal doses of A/PR8, and the concentrations of cytokines and the population of innate immune cells were analyzed in BALF and lungs at 3 days post-infection (d.p.i). The levels of TNF-α, IFN-γ, CCL2, and CXCL1 were significantly lower in BLM-treated mice than those in PBS-treated mice following A/PR8 infection. There was no significant change in the level of IL-6 (**Figure 2A**), whereas the level of IL-12p40 increased in BLM-treated mice than those in PBS-treated mice (**Figure 2A**). The infiltration of neutrophils in the lung was also significantly decreased in BLM-treated mice compared with those in PBS-treated mice; however, the infiltration of monocytes and the number of tissue resident alveolar macrophages were comparable between BLM-treated mice and PBS-treated mice (**Figure 2B**). Collectively, these results suggest that pretreatment of mice with BLM attenuates influenza-mediated lung inflammation and induces resistance to influenza infection in mice.

### BLM Treatment Exerts an Antiviral Effect Through Type I IFN Receptor Signaling

Since BLM treatment increased the expression of IL-12p40, a subunit of IL-12p70 (20), which activates natural killer (NK) cells and cytotoxic CD8^+^ T lymphocytes (21, 22), we then confirmed that these cells are associated with controlling influenza infection. We used depletion antibodies to remove both NK and CD8^+^ T cells, although this did not significantly affect the susceptibility of BLM-treated mice to A/PR8 infection (**Supplementary Figure 2**). These results indicate that although NK cells and CD8^+^ T cells are important immune cells for controlling viral infection, BLM-mediated attenuation of influenza infection is not due to their increased activity.

**Figure 3:**
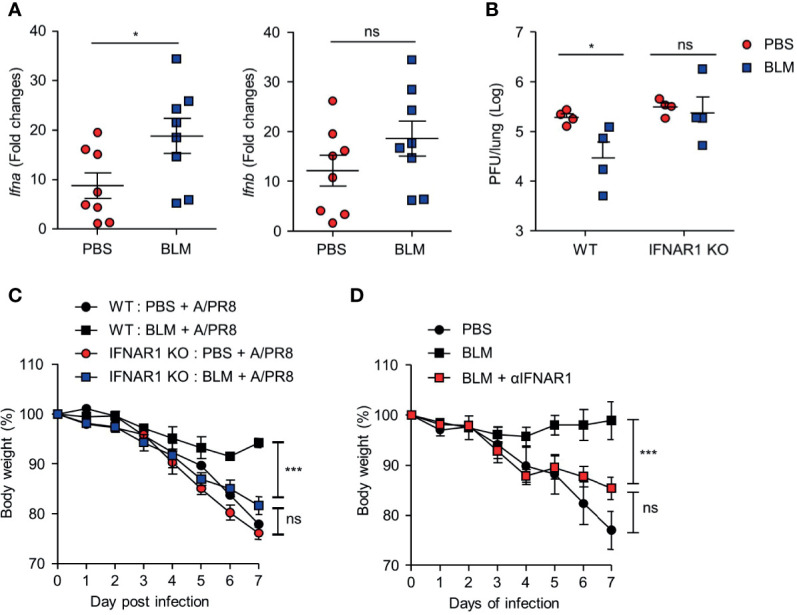
BLM treatment enables antiviral effects *via* an IFN-α-dependent manner. BLM-administered mice were intranasally infected with lethal doses and sacrificed after 1 or 2 days post infection (d.p.i.) **(A)** Measurement of host *Ifna4* and *Ifnb1* mRNA at 1 d.p.i. by qRT-PCR (●, n = 8; ■, n = 8). **(B)** Viral activity of lung tissue from BLM-treated wild type (WT) and IFNAR1^-/-^ mice infected with a lethal dose at 2 d.p.i. using the A549 cell line (●; n = 4, ■; n = 4). **(C)** BLM-treated WT and IFNAR1^-/-^ mice were infected with 1 × 10^3^ PFU and body weight was monitored for 7 days (●, n = 2; ■, n = 2; ●;, n = 6; ■, n = 8). **(D)** αIFNAR1 was administered at -1, 2, and 5 d.p.i., and mice were infected with sub-lethal virus dose, and body weight monitored for 7 days (●, n = 3; ■, n = 3; ■, n = 4). Data are expressed as mean ± SEM of representative of 1 experiment for **(B, D)** and 2 independent experiments for **(A, C)**. Statistical analysis was performed for **(A, B)** using t-tests and in **(C, D)** by Two-way ANOVA (*p < 0.05, ***p < 0.001). ns, not significant.

IFN-I is a major cytokine that inhibits viral replication by inducing the expression of ISGs (23). To assess whether BLM treatment affects IFN-I expression, IFN-I transcription was analyzed in lung tissues of BLM- and PBS-treated mice 1 day after infection with a lethal dose of A/PR8. The level of *Ifna4* mRNA expression was higher in BLM-treated mice than that in PBS-treated mice. In contrast, there were no significant differences in *Ifnb1* transcription between PBS- and BLM-treated mice (**Figure 3A**).

We then used IFNAR1^-/-^ knockout mice to confirm the role of IFN-I in antiviral effects after BLM treatment. Ablation of IFN-I signaling nullified the effect of BLM pretreatment on antiviral resistance as the pulmonary influenza burden was increased following A/PR8 infection (**Figure 3B**). In addition, the attenuation of A/PR8 infection-mediated weight loss was reduced in IFNAR1^-/-^ mice in the BLM-treated group (**Figure 3C**). Similarly, treatment with IFNAR1 blocking antibody (αIFNAR1) in BLM-pretreated mice infected with sub-lethal doses of A/PR8 showed reduced body weight for 7 days, suggesting that BLM treatment did not inhibit influenza infection (**Figure 3D**). These results suggest that BLM pretreatment attenuates influenza virus infection by activating IFN-I signaling in mice.

### BLM Treatment Increased dsDNA Which Activates the cGAS-STING Pathway for IFN-I Production

**Figure 4:**
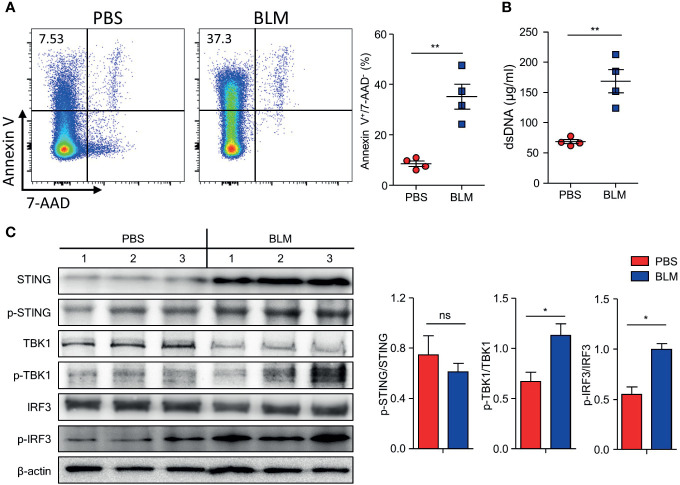
Bleomycin (BLM)-induced lung injury activates cGAS-STING pathway. Mice were administered BLM intranasally, and the lungs were analyzed after 7 days. **(A)** Apoptotic cells in BALF were measured using FACS (■, n = 4; ■, n = 4). **(B)** dsDNA in BALF was quantified (■, n = 4; ■, n = 4). **(C)** Immunoblots of STING, p-STING, TBK1, p-TBK, IRF3, p-IRF3, and β-actin expression in lung homogenates and normalized expression of each of phosphorylated form to its total form indicated (PBS, n = 3; BLM, n = 3). Data are expressed as ±SEM and representative of 1 experiment. Statistical analysis was performed using t-tests (*p < 0.05, **p < 0.01). ns, not significant.

BLM treatment induces apoptosis in alveolar epithelial cells and leads to the release of apoptosis-induced DNA fragments (24, 25). Increased dsDNA binds to cGAS and activates the STING pathway, which phosphorylates IRF-3 to produce IFN-I (26). We hypothesized that BLM-induced self-DNA leakage activates the cGAS-STING pathway, which results in enhanced IFN-I expression in response to further stimuli. BLM treatment induced apoptosis of cells and increased dsDNA in the BALF (**Figures 4A, B**). The levels of proteins downstream of the cGAS-STING pathway were assessed in lung tissues of BLM- and PBS-treated mice. Although the BLM treatment did not changed the ratio of phosphorylated (p)-STING to total STING, expression of STING, p-STING, p-TBK1, and p-IRF3 in lung tissue was clearly elevated, suggesting that BLM treatment activates the cGAS-STING pathway (**Figure 4C**).

**Figure 5:**
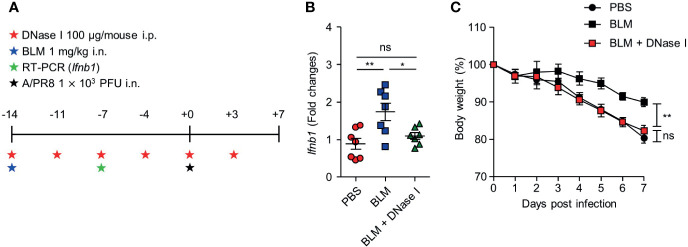
DNase I treatment reduces IFN-I transcription and resistance to influenza virus infection in BLM-treated mice. **(A)** Mice were administered BLM intranasally and DNase I intraperitoneally following the scheme. ****(B) Gene expression of *Ifnb1* in lung was analyzed (●, n = 7; ■, n =7; ▲, n=7). **(C)** Body weight was monitored for 7 days (●, n = 6; ■, n = 6; ■, n = 8). Data are expressed as ±SEM and representative of 2 independent experiments. Statistical analysis was performed with One-way ANOVA for **(B)** and Two-way ANOVA for **(C)** (*p < 0.05, **p < 0.01). ns, not significant.

To assess whether dsDNA-induced cGAS-STING pathway enhanced IFN-I expression, the mice were treated with DNase I after BLM treatment (**Figure 5A**). BLM-treated mice showed increased *Ifnb1* transcription at 7 days after treatment but co-treatment with DNase I suppressed *Ifnb1* transcription to the level of PBS-treated control mice (**Figure 5B**). This observation suggests that sensing the DNA in BLM treatment mice may protect mice by inducing IFN-I. Indeed, BLM and DNase I-co-treated mice showed significantly decreased body weight compared to that of BLM control mice (**Figure 5C**).

### pDCs Are Required for BLM-Mediated Antiviral Activity

**Figure 6:**
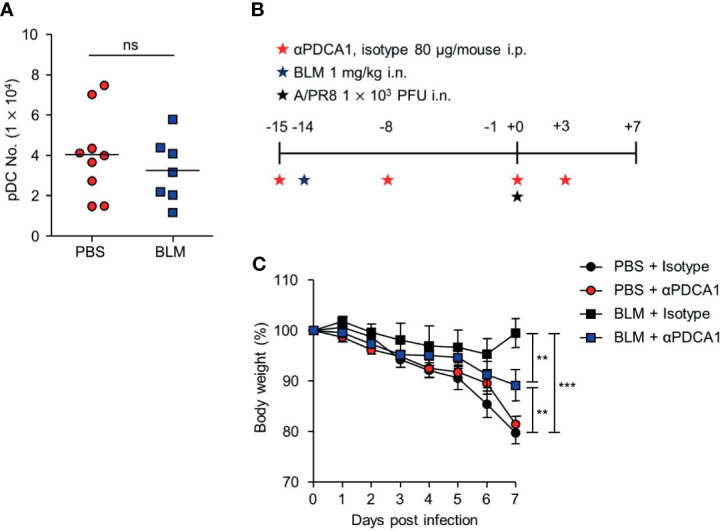
pDCs contribute an antiviral effect after BLM-induced acute lung injury. Mice were sacrificed 14 days after BLM treatment. **(A)** Analysis of the number of pDCs in the lung by FACS (●, n = 9; ■, n = 7). **(B)** αPDCA-1 and isotype were administered intraperitoneally (i.p.) to mice following the scheme; mice were then infected with a sub-lethal virus dose intranasally (i.n.). **(C)** Body weight was monitored for 7 days (●, n = 5; ●, n = 6; ■, n = 4, ■; n = 5). Data are expressed as mean ±SEM and representative of 2 independent experiments. Results in **(A)** were analyzed statistically using t-tests and in **(B)** using Two-way ANOVA (**p < 0.01, ***p < 0.001). ns, not significant.

**Figure 7:**
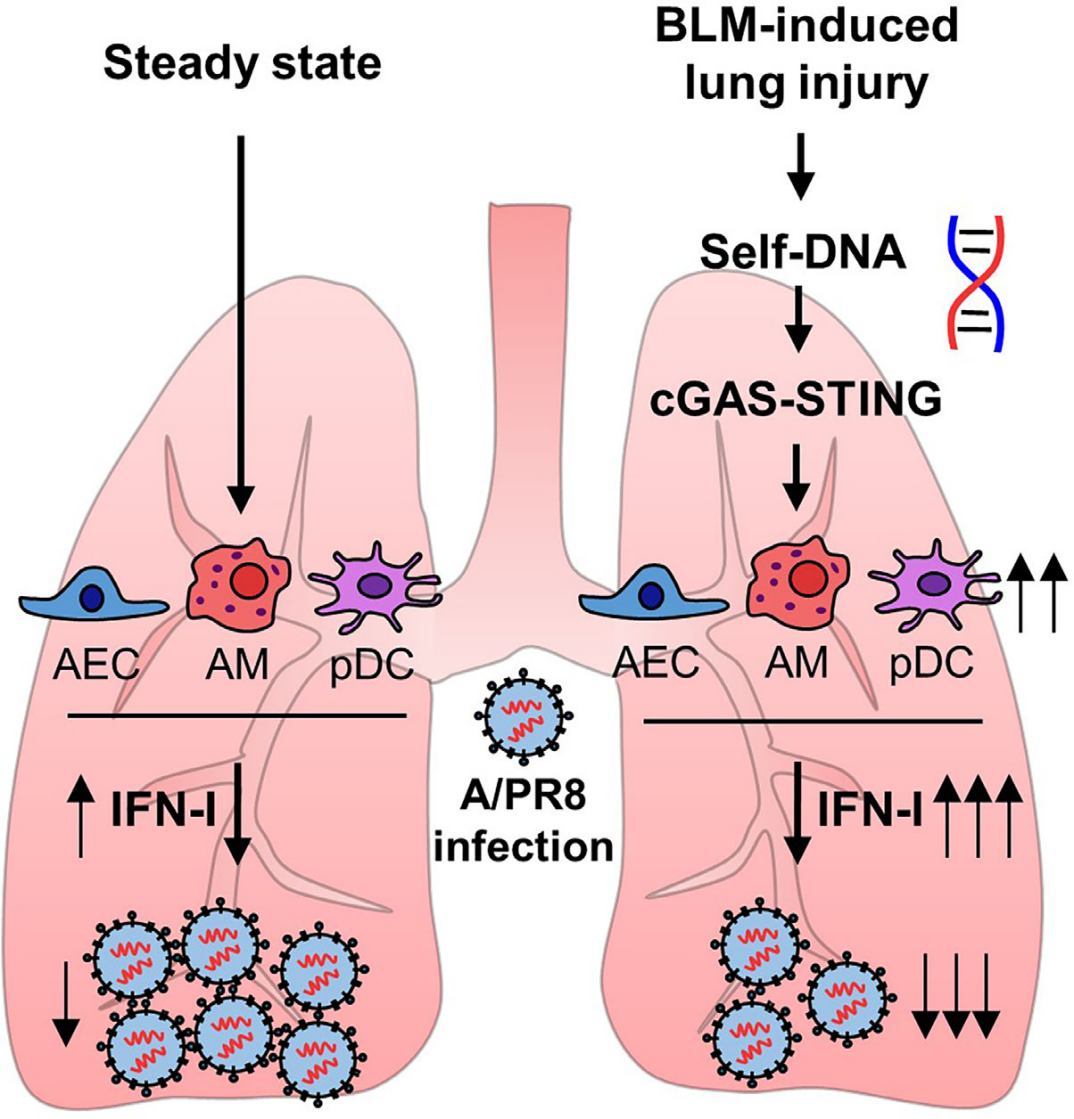
Bleomycin-induced lung injury releases self-DNA that activates cGAS-STING pathway. Activated cGAS-STING pathway affects to enhanced IFN-I transcription, and pDCs are responsible for increased antiviral effects.

Alveolar epithelial cells, macrophages, and pDCs in the lungs have been reported to produce IFN-I following infection with influenza virus (27). pDCs are the major source of IFN-I in response to viral infection or dsDNA (28). Thus, we assumed that pDCs may up-regulate their activity by sensing self-DNA *via* the cGAS-STING pathway (29). Although the total number of pDCs was comparable in the lungs of mice obtained from 14 days after BLM or PBS treatment (**Figure 6A**), the depletion of pDCs using anti-PDCA1 Ab substantially reduced resistance to A/PR8 infection in BLM-treated mice (**Figures 6B, C**). Overall, these results indicate that BLM treatment stimulates IFN-I secretion in pDCs to enhance resistance to influenza virus infection (**Figure 7**).

## Discussion

Infection with influenza can damage alveolar epithelial cells, induce ALI, and result in chronic inflammation and pulmonary fibrosis (4, 30). However, it is unclear whether influenza virus infection is detrimental to patients with ALI and advanced idiopathic pulmonary fibrosis (IPF). Influenza virus has been reported to aggravate IPF symptoms (31, 32), whereas other studies found that the influenza virus was undetectable or asymptomatic in patients with IPF (33–35). The results reported here suggest that asymptomatic or insignificant viral infection in IPF patients is due to the enhanced antiviral immunity conferred by increased IFN-I signaling.

DNA sensors, including cGAS-STING and TLR9, are crucial for IFN-I-dependent antiviral responses (36, 37). In a pulmonary injury model, the cGAS-STING pathway was reported to play a more prominent role than that of TLR9 in the IFN-I response (6, 38). DNA fragments leaked from damaged tissues activate STING signaling, leading to the activation of the transcription factors IRF3 and NF-κB, which are essential for IFN-I production (39). Indeed, silica-induced injury results in self-DNA-mediated IFN-I production in the lungs (6, 7). However, another study using a cisplatin-induced renal fibrosis model showed that STING induced NF-κB expression rather than that of IFN-I in an IRF3-independent manner (40). In addition, tubular cell lines exposed to cisplatin showed phosphorylation of TBK1 and p65 but not that of IRF3 (40). The present study confirmed that BLM-induced pulmonary injury employed activation of IRF3 downstream of STING activation. Combined with the results obtained in the silica-induced lung injury model, these findings indicated that ALI induced by BLM activated STING/TBK1/IRF3 signaling, resulting in the production of IFN-I. Increased ISGs are detected in tracheal samples of children with ARDS and IPF patients (41, 42). Although the level of cGAS is increased in IPF patients who have severe inflammation, the association of STING/TBK1/IRF3 signaling in IPF disease prognosis is not clearly reported (42). Activation of a number of pathogen recognition pathways could increase STING production. TLR9 is well known for recognizing self-DNA as well (43–47) that might be related with increased STING production. Further study is required to elucidate whether TLR9 and STING signaling are associated with increasing STING production, which could explain the STING and pSTING levels.

pDCs respond to pathogen-associated molecular patterns and mainly produce IFN-I, triggering inflammation and immune tolerance (28). pDCs also restrict viral replication by modulating lipid biosynthesis or metabolism (48, 49). IFN-I modulates lipid metabolism by increasing 2,5-hydroxycholesterol, which inhibits viral replication (48, 50). pDC-derived IFN-I also enhances fatty acid oxidation in non-hematopoietic cells that repress A/PR8 replication (51). These findings indicate that various IFN-I-related functions are generally responsible for the antiviral effects of pDCs. Although depletion of pDCs resulted in a partial loss of protective antiviral effect, this may have been due to other cell types that compensated for IFN-I production after pDC depletion. While the influenza virus expresses non-structural 1 (NS1) protein to counteract IFN-I and escape lipid-dependent antiviral effects (52), the present study suggests that pDC-derived IFN-I efficiently suppresses the replication of influenza virus in BLM-induced ALI.

In general, cytotoxic lymphocytes are crucial for the removal of invading viruses (53). However, in the present study, we found that depletion of CD8^+^ T cells and NK cells had little effect on viral clearance. Since induced IFN-I-controlled early virus infection is more efficient in BLM-treated lungs, the role of cytotoxic lymphocytes may be less prominent. Although CD8^+^ T cells and NK cells were not required for the antiviral effects of BLM, recruitment of these cells was significantly enhanced in the lungs of BLM-treated mice. CD8^+^ T cells and NK cells are the lymphocytes most prominently recruited to the lungs to attenuate BLM-induced pulmonary inflammation (54, 55). Recruited lymphocytes stimulate fibroblasts to express more collagen and contribute to tissue restoration (56). However, the depletion of CD8^+^ T cells or NK cells did not significantly alter the antiviral effect of BLM. Thus, the increased level of IL-12p40 in BALF after BLM treatment might be associated with the tissue repair since IL-12p40 is one of subunits of IL-23. IL-23 promotes differentiation of naïve CD4^+^ T cells to TH17 (20) which produces diverse cytokines such as IL-17, IL-17F, IL-21 and IL-22. Among them, especially, IL-17 promotes inflammation and fibrosis to recover damaged tissue (57). It also increases TGF-β, the master cytokine that causes lung fibrosis (58).

Type III interferon (IFN-λ) is transcribed along with IFN-I upon phosphorylation of IRF3 or IRF7, then binds to its receptor IFNLR1/IL-10R2 that activates IRF9 to induce ISGs by phosphorylating STAT1 and STAT2 as IFN-I does (59). Although IFN-λ shares the signaling cascade to express ISGs with IFN-I, IFN-λ has distinct role in controlling viral infection. IFN-λ is reported to regulate influenza virus A infection by increasing basal ISGs gene expression in the absence of IFNAR1 and also induces resistance to sub-lethal influenza virus A infection by attenuating IFN-I-induced excessive inflammation (60, 61). In the current study, although IFN-λ could also be associated with the increased antiviral effects by BLM, the blockade of IFANR1 in BLM-treated mice showed decrease in BLM-induced viral resistance. However, further study is required to identify the role of IFN-λ in BLM-induced viral resistance.

Overall, these findings suggest that patients with ALI-induced ARDS show attenuated symptoms in response to pulmonary virus infection. In this respect, drug candidates that utilize dsDNA to activate the STING/TBK1/IRF3 pathway may be promising for controlling pulmonary virus infection.

## Data Availability Statement

The original contributions presented in the study are included in the article/**Supplementary Material**. Further inquiries can be directed to the corresponding authors.

## Ethics Statement

The animal study was reviewed and approved by Institutional Animal Care and Use Committee of Kangwon National University (Permit Number: KW-200131-2).

## Author Contributions

S-US, J-HJ, and M-NK designed this study. J-HJ performed and analyzed experiments. J-HJ, S-US, and H-JK wrote the manuscript. B-SB, J-MC, YSC performed data curation. All authors contributed to the article and approved the submitted version.

## Funding

This work was supported by a National Research Foundation of Korea (NRF) grant 2016R1C1B2008089 (S-US); NRF-2019M3C9A6082487, NRF-2020R1A2B5B03001450, NRF-2017M3A9C8060390, and NRF-2020R1A2B5B02001552 (H-JK); NRF-2020R1A2B5B03001450 (M-NK).

## Conflict of Interest

The authors declare that the research was conducted in the absence of any commercial or financial relationships that could be construed as a potential conflict of interest.

## Publisher’s Note

All claims expressed in this article are solely those of the authors and do not necessarily represent those of their affiliated organizations, or those of the publisher, the editors and the reviewers. Any product that may be evaluated in this article, or claim that may be made by its manufacturer, is not guaranteed or endorsed by the publisher.
