# Supplementary material for: Bleomycin-Induced Lung Injury Increases Resistance to Influenza Virus Infection in a Type I Interferon-Dependent Manner
Source: Front Immunol. 2021 Aug 18;12:697162. doi: 10.3389/fimmu.2021.697162 (PMC8416411; doi:10.3389/fimmu.2021.697162)
Supplement: Supplementary file 1 [file DataSheet_1.pdf]

Supplementary Figure 1.

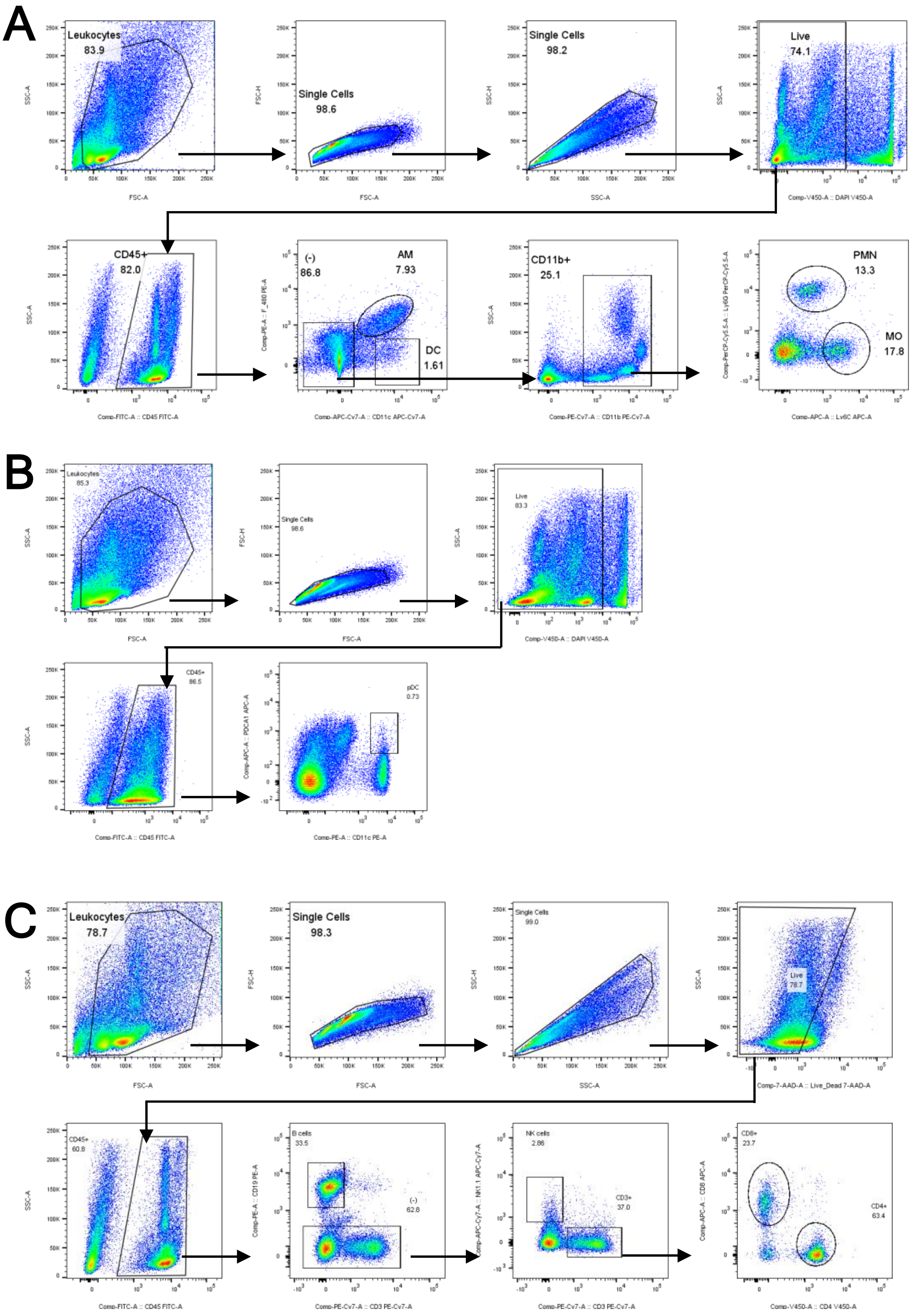

Supplementary Figure 1. Gating strategies for (A) figure 2, (B) figure 6 and (C) Supplementary figure 2 are indicated.

Supplementary Figure 2.

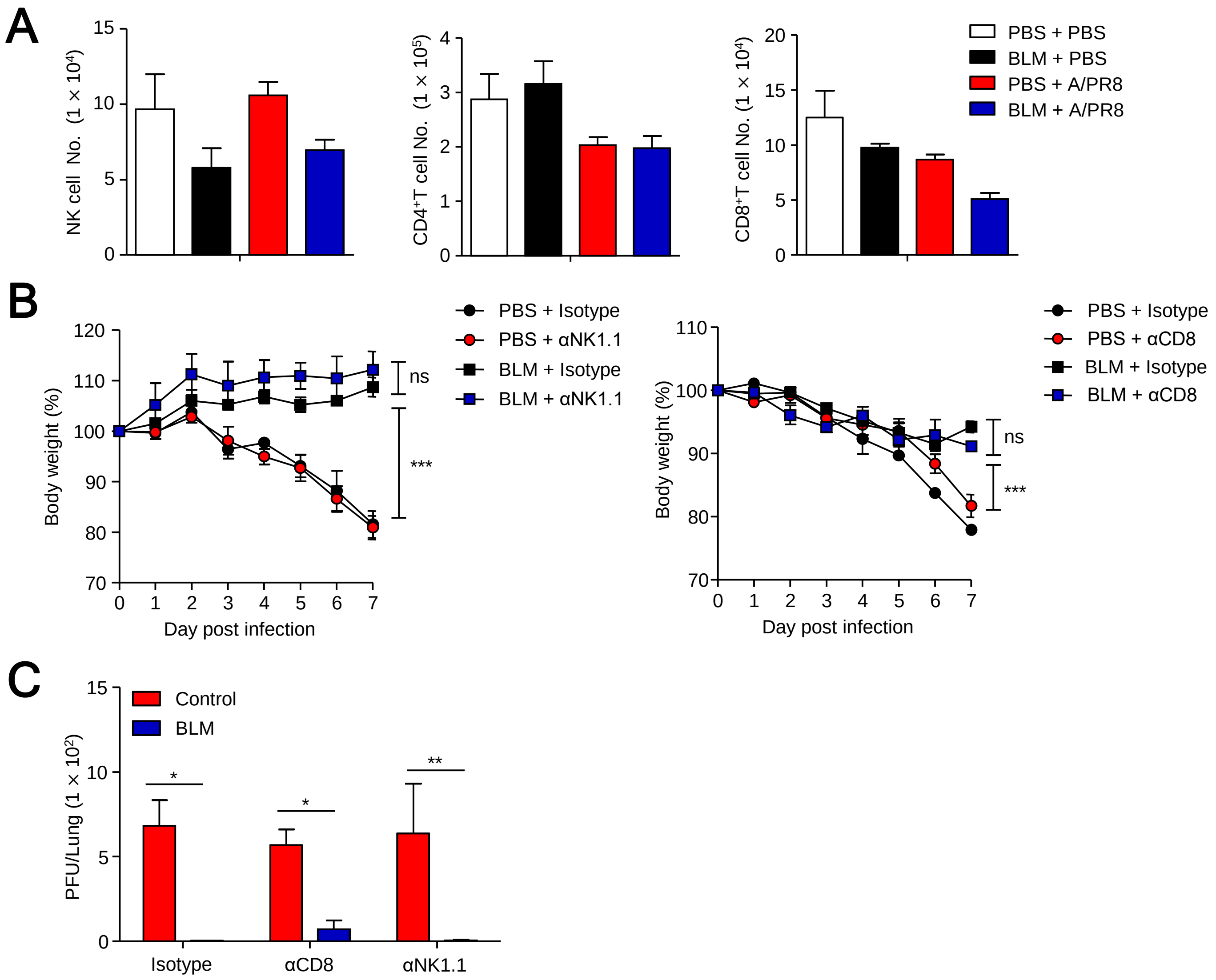

Supplementary Figure 2. Cytotoxicity lymphoid cells were not associated with controlling influenza virus infection. BLM-treated mice were infected intranasally with sub-lethal dose of virus and euthanized 3 days later. (A) FACS analysis of the numbers of NK and $CD8^+$ T cells in the lungs. (B) NK cells and $CD8^+$ T cells were depleted from mice by intraperitoneal antibody administration at 8, 4, and 1 day before A/PR8 infection, and body weight was monitored for 7 days. (C) At 7 d.p.i., mouse lungs were extracted, and the PFU assay was performed using the A549 cell line to titrate A/PR8. Data are expressed as the ± SEM of representative of 1 experiment. Statistical analysis of data was performed using ANOVA (*$p < 0.05$, **$p < 0.01$, ***$p < 0.001$). ns, not significant.
